# Successful Management of Angiotensin-Converting Enzyme Inhibitor-Induced Angioedema With Tranexamic Acid: A Case Report

**DOI:** 10.7759/cureus.98001

**Published:** 2025-11-28

**Authors:** Mussab Ibrahem

**Affiliations:** 1 Emergency Medicine, Glangwili General Hospital, Carmarthen, GBR

**Keywords:** ace inhibitor-induced angioedema, bradykinin-induced angioedema, bradykinin-mediated angioedema, facial angioedema, tranexamic acid (txa)

## Abstract

Angioedema is a potentially life-threatening emergency that can present with facial swelling and airway compromise. When induced by angiotensin-converting enzyme (ACE) inhibitors (ACEIs), the mechanism is bradykinin-mediated, rendering conventional anaphylaxis therapies largely ineffective. We report a case of a 68-year-old male who presented to the Emergency Department with acute, progressive facial and tongue swelling, sore throat, and mild dyspnoea, consistent with moderate (Grade II) ACEI-induced angioedema. His history included hypertension treated with a recently up-titrated dose of ramipril, type 2 diabetes, gout, and prior deep vein thrombosis. Initial management following anaphylaxis protocol with intramuscular adrenaline, intravenous hydrocortisone, chlorphenamine, and adrenaline nebulisers produced no improvement. Given the strong suspicion of bradykinin-mediated angioedema, 1 g of intravenous tranexamic acid (TXA) was administered. Marked improvement was observed within 30 minutes, with complete resolution of swelling within two hours. The patient was admitted overnight for observation, ramipril was discontinued, and he remained symptom-free at one-year follow-up. This case highlights TXA as a safe, effective, and accessible therapeutic option for ACEI-induced angioedema, particularly in settings where targeted therapies are unavailable. It underscores the importance of recognizing this distinct clinical entity and the need for further prospective studies to define TXA's role in treatment algorithms.

## Introduction

Angioedema is characterized by rapid, localized, and transient swelling of the deep dermis, subcutaneous, and submucosal tissues due to a sudden increase in vascular permeability [1]. This condition can be life-threatening when it involves the upper airway. A significant and distinct subtype is angiotensin-converting enzyme (ACE) inhibitor-induced angioedema (ACEI-AE), which is estimated to account for up to 30% of all angioedema cases presenting to emergency departments [2,3]. Among the millions of patients prescribed ACEIs for conditions such as hypertension and heart failure, the reported prevalence of this adverse effect ranges from 0.1% to 0.7%, though underreporting suggests that the true incidence may be higher [4].

The pathophysiology of ACEI-AE is fundamentally different from the more common histamine-mediated allergic angioedema. ACEIs block the degradation of bradykinin, a potent vasoactive peptide, leading to its accumulation [5]. Elevated bradykinin levels cause vasodilation and capillary leakage, resulting in tissue swelling. This bradykinin-mediated mechanism explains why standard therapies for anaphylaxis-adrenaline, corticosteroids, and antihistamines-are typically ineffective for ACEI-AE [6].

Clinically, ACEI-AE typically presents with asymmetric, non-pitting, and non-pruritic swelling of the lips, tongue, face, and sometimes the upper airway. While it can occur shortly after initiating the drug, a hallmark feature is its unpredictable onset, which may be delayed for months or even years after uneventful ACEI use [7]. The primary management goals are immediate airway protection and discontinuation of the offending drug. However, the absence of universally approved, effective first-line pharmacological treatments poses a significant clinical challenge. Targeted agents such as icatibant (a bradykinin B2 receptor antagonist) and C1-esterase inhibitor concentrate have demonstrated efficacy in reducing symptom duration but are prohibitively expensive and often not stocked in many emergency departments, especially in resource-limited settings [8].

Tranexamic acid (TXA), a synthetic antifibrinolytic agent, has emerged as a potential therapeutic alternative. Its mechanism in this context is believed to be the inhibition of plasminogen activation to plasmin. Since plasmin is a key activator of the kallikrein-kinin system, TXA indirectly reduces the production of bradykinin [9,10]. Given its wide availability, low cost, and excellent safety profile, TXA represents a pragmatic option for emergency physicians. This case report details the successful use of TXA in a patient with ACEI-AE that was unresponsive to conventional measures, highlighting its practical utility and contributing to the growing literature on its application for this condition.

## Case presentation

A 68-year-old man presented to the Emergency Department at 23:30 on 03/04/2024 with a four-hour history of progressive swelling of the lips and tongue associated with mild dyspnoea and sore throat. He associated this with a subjective sensation of a sore throat and mild dyspnoea. He explicitly denied any urticaria, pruritus, wheezing, rash, or exposure to new foods, medications, or other potential allergens.

His significant past medical history included hypertension, type 2 diabetes mellitus, gout, and a previous episode of deep vein thrombosis. His medication list consisted of ramipril 10 mg daily (a dose that had been increased from 5 mg approximately six weeks prior), metformin 500 mg twice daily, allopurinol 100 mg daily, and rivaroxaban 20 mg daily. He reported no known drug allergies.

On initial examination, the patient was alert and able to maintain his own airway. He had notable macroglossia and swelling of the lips. His phonation was mildly dysarthric, but there was no stridor, drooling, or other signs of imminent airway obstruction. The absence of urticaria or other skin findings was confirmed. His vital signs and initial laboratory investigations were within normal limits, as detailed in Table 1. The clinical presentation was consistent with moderate severity, Grade II angioedema, characterized by visible tissue swelling with mild, subjective respiratory symptoms but no objective signs of airway compromise [11].

**Table 1:** Patient's vital signs and initial laboratory results on presentation.

Parameter	Patient Value	Reference Range	Units
Temperature	36.3	36.0 – 37.5	°C
Heart Rate	76	60 – 100	beats per minute
Blood Pressure	167/93	100-140 / 60-90	mmHg
Respiratory Rate	19	12 – 20	breaths per minute
Oxygen Saturation	96	≥ 94	% (room air)
Haemoglobin	142	130 – 170	grams per litre
White Cell Count	7.8	4.0 – 11.0	x 10⁹ per litre
Platelets	255	150 – 400	x 10⁹ per litre
Urea	5.2	2.5 – 7.8	millimoles per litre
Creatinine	88	60 – 110	micromoles per litre
C-reactive Protein	<5	<10	milligrams per litre
Glucose	7.1	4.0 – 7.8	millimoles per litre

The patient was initially managed according to the local anaphylaxis protocol due to the potential for airway compromise. This included the administration of 500 micrograms of intramuscular adrenaline, 200 milligrams of intravenous hydrocortisone, and 10 milligrams of intravenous chlorphenamine at 23:50. This was followed by a nebulised dose of adrenaline (1 mg) and 500 millilitres of intravenous 0.9% saline. After 30 minutes of observation, there was no discernible improvement in the swelling or his symptoms.

Given the lack of response to standard therapy and the classic presentation in the context of ACEI use, a diagnosis of bradykinin-mediated ACEI-AE was strongly suspected. After consultation with the on-call anaesthetics team for airway standby, a decision was made to administer 1 gram of intravenous TXA over 10 minutes at 02:15. The patient began to report subjective improvement approximately 15 minutes after the TXA infusion was completed. By the 30-minute mark, a marked objective reduction in tongue and lip swelling was evident. The swelling resolved completely within two hours of TXA administration. The patient tolerated the procedure well with no adverse effects. A mast cell tryptase level, checked after clinical improvement, was 6 µg/L, which is within the normal range, further supporting a non-allergic, bradykinin-mediated etiology.

The patient was admitted to a monitored bed for overnight observation. Ramipril was permanently discontinued and replaced with bendroflumethiazide 2.5 mg daily for hypertension management. He was discharged the following day in a stable condition. At follow-up appointments two weeks and one year later, he reported no recurrence of angioedema.

Note: No clinical photographs are available for this case, as they were not obtained during the acute management in the emergency setting.

## Discussion

This case provides a compelling example of the diagnostic and therapeutic challenges posed by ACEI-induced angioedema and demonstrates the potential role of a readily available agent, TXA. The patient's presentation was classic for ACEI-AE: asymmetric orofacial swelling without urticaria, occurring in the context of recent ACEI dose escalation. The failure to respond to adequate doses of adrenaline, corticosteroids, and antihistamines is a hallmark of bradykinin-mediated pathology and should prompt clinicians to consider this diagnosis [6]. The normal mast cell tryptase level post-recovery provides further biochemical support that this was not a mast cell-driven allergic reaction.

The pathophysiology of ACEI-AE centers on the accumulation of bradykinin. ACE, also known as kininase II, is a primary enzyme responsible for degrading bradykinin. Its inhibition leads to elevated bradykinin levels, which activate the B2 receptor on endothelial cells, causing profound vasodilation and vascular leakage [5]. TXA is thought to interrupt this cascade upstream by potently inhibiting the conversion of plasminogen to plasmin. Plasmin is a key activator of factor XII and a potent stimulator of kallikrein formation, which in turn generates bradykinin from high-molecular-weight kininogen. By suppressing plasmin formation, TXA indirectly reduces bradykinin production [9].

This postulated mechanism is supported by a growing number of clinical reports. For instance, Beauchêne et al. described successful first-line use of TXA in bradykinin-mediated angioedema with rapid resolution of symptoms [10]. However, the evidence is not entirely consistent. A recent large retrospective multicenter study by Lindauer et al. found that, while TXA was frequently used, its administration was associated with a longer emergency department length of stay and higher ICU admission rates, though the authors noted significant confounding by severity, as patients with more severe angioedema were more likely to receive TXA [12]. This underscores the critical need for prospective, randomized trials to definitively establish its efficacy.

The current management of ACEI-AE in many centers remains supportive, with specific, targeted therapies such as icatibant and C1-esterase inhibitor concentrate often reserved for severe or refractory cases due to their high cost and limited availability [8]. In this context, TXA presents a valuable intermediate option. It is inexpensive and widely available and has an excellent safety profile, well-documented in large trials such as CRASH-2 and WOMAN, for other indications [13,14]. Its use can be a crucial intervention, particularly in resource-limited settings or while awaiting the arrival of more specific agents.

Several important lessons can be drawn from this case. First, clinician awareness is paramount; ACEI-AE can occur after years of uneventful therapy, and its recognition is the first step toward appropriate management. Second, the initial use of anaphylaxis protocols is not incorrect when the diagnosis is uncertain, but a lack of response should trigger a swift change in therapeutic strategy. Finally, while our case and others suggest TXA is effective, it is vital to avoid overstating its role. Our positive outcome must be interpreted within the limitations of a single case report, which cannot establish causality or prove efficacy over spontaneous resolution. The patient's improvement was temporally linked to TXA administration, but larger, controlled studies are needed to define its precise place in the treatment algorithm, including optimal dosing and timing.

## Conclusions

This report describes the successful management of moderate (Grade II) ACEI-induced angioedema with intravenous TXA after the failure of conventional anaphylaxis medications. The rapid and complete resolution of symptoms following TXA administration highlights its potential as a safe, effective, and accessible therapeutic alternative, especially in emergency departments where targeted therapies are not readily available. This case reinforces the importance of differentiating bradykinin-mediated angioedema from its histaminergic counterpart to guide appropriate treatment. While promising, these findings warrant validation through prospective, controlled clinical trials to determine the optimal role and protocol for TXA use in this potentially life-threatening condition.
